# Quantitative Proteomics Identifies Novel Nrf2-Mediated Adaptative Signaling Pathways in Skeletal Muscle Following Exercise Training

**DOI:** 10.3390/antiox12010151

**Published:** 2023-01-09

**Authors:** Anjali Bhat, Rafay Abu, Sankarasubramanian Jagadesan, Neetha Nanoth Vellichirammal, Ved Vasishtha Pendyala, Li Yu, Tara L. Rudebush, Chittibabu Guda, Irving H. Zucker, Vikas Kumar, Lie Gao

**Affiliations:** 1 Department of Anesthesiology, University of Nebraska Medical Center, Omaha, NE 68198, USA; 2 Mass Spectrometry and Proteomics Core Facility, University of Nebraska Medical Center, Omaha, NE 68198, USA; 3 Department of Biochemistry, Glocal University, Saharanpur 247121, Uttar Pradesh, India; 4 Department of Genetics, Cell Biology & Anatomy, University of Nebraska Medical Center, Omaha, NE 68198, USA; 5 Department of Cellular & Integrative Physiology, University of Nebraska Medical Center, Omaha, NE 68198, USA

**Keywords:** Nrf2, skeletal muscle, exercise, proteomics

## Abstract

Exercise training (ExT) improves skeletal muscle health via multiple adaptative pathways. Nrf2 is a principal antioxidant transcription factor responsible for maintaining intracellular redox homeostasis. In this study, we hypothesized that Nrf2 is essential for adaptative responses to ExT and thus beneficial for muscle. Experiments were carried out on male wild type (WT) and iMS-*Nrf2^flox/flox^* inducible muscle-specific Nrf2 (KO) mice, which were randomly assigned to serve as sedentary controls (Sed) or underwent 3 weeks of treadmill ExT thus generating four groups: WT-Sed, WT-ExT, KO-Sed, and KO-ExT groups. Mice were examined for exercise performance and in situ tibialis anterior (TA) contractility, followed by mass spectrometry-based proteomics and bioinformatics to identify differentially expressed proteins and signaling pathways. We found that maximal running distance was significantly longer in the WT-ExT group compared to the WT-Sed group, whereas this capacity was impaired in KO-ExT mice. Force generation and fatigue tolerance of the TA were enhanced in WT-ExT, but reduced in KO-ExT, compared to Sed controls. Proteomic analysis further revealed that ExT upregulated 576 proteins in WT but downregulated 207 proteins in KO mice. These proteins represent pathways in redox homeostasis, mitochondrial respiration, and proteomic adaptation of muscle to ExT. In summary, our data suggest a critical role of Nrf2 in the beneficial effects of SkM and adaptation to ExT.

## 1. Introduction

Physical activity is a fundamental component of human health [1]. Exercise training (ExT) effectively prevents, treats, or reverses many pathological conditions in vital organs from the brain to the kidney [2,3]. Skeletal muscle (SkM) provides contractile force for body movement and exhibits functional enhancement following physical activity. Repetitive contraction–relaxation significantly impacts muscular metabolism, function, and structure by inducing mitochondrial biogenesis, hyperplasia, hypertrophy, fiber type transformation, and angiogenesis [4]. These phenotypic changes are the result of an array of gene expressions and intracellular signaling transduction pathways in response to contraction-evoked biophysical and biochemical stimuli among which excess reactive oxygen species (ROS) play a major role [5]. Excess ROS oxidize cellular components, altering their native structure which can affect their homeostatic function. On the other hand, low levels of ROS serve as physiological messengers essential for activating intracellular signaling pathways under normal conditions (oxidative eustress) [6]. The level of ROS depends on the balance between production of ROS and the ability of the cell to scavenge ROS by antioxidant proteins. Consequently, oxidative distress occurs when excess ROS accumulates. However, when cellular ROS is diminished to below physiological levels and their signaling function is perturbed, additional pathological conditions associated with redox biology, known as ‘reductive stress’, are prevalent [7]. It is imperative for healthy cells to maintain a delicate balance between ROS generation and elimination.

Nuclear factor-erythroid factor 2-related factor 2 (Nrf2) is a crucial master transcription factor, affecting nearly 500 genes that encode proteins associated with redox equilibrium, energy metabolism, drug detoxification, and stress responses [8,9,10]. The function of Nrf2 is strictly regulated by its negative regulator Keap1, a protein classified as BTB-Kelch family, which binds to Cullin 3 and Rbx1 to form a multisubunit Cullins-RING ligase for protein ubiquitination. Two C-terminal Kelch-domains of the Keap1 dimer separately bind to the ETGE and DLG motifs of Nrf2 Neh2-domain, whose lysine residues are therefore ubiquitinylated by Cullin ligase, labeling Nrf2 for proteasomal degradation [11]. This negative regulatory process is dependent on ROS-induced modification of three sensor cysteines (Cys151/Cys273/Cys288) in Keap1. These perturbations lead to Nrf2-mediated antioxidant defense activation triggered by DNA binding to antioxidant response elements (AREs) of appropriate genes [12]. Together, ROS and the Nrf2/Keap1 system form a delicate feedback regulatory mechanism critical for the maintenance of intracellular redox homeostasis.

To investigate the functional significance of Nrf2 in SkM, we created two transgenic mouse lines—iMS-*Nrf2^flox/flox^* and iMS-*Keap1^flox/flox^*—which allowed us to selectively delete (i.e., Nrf2 gene knockout) or upregulate (i.e., Keap1 gene knockout) SkM Nrf2 in a Tamoxifen-inducible manner [13]. Utilizing label-free mass spectrometry-based proteomic profiling of these mice, we identified over 200 cytoprotective proteins that were upregulated and four intracellular signaling pathways activated in SkM by Nrf2 [13]. We further explored the redox changes by employing iodoacetyl tandem mass tag (iodoTMT^TM^)-labeled cysteine quantitation. We found 34 redox sensitive proteins harboring reversible cysteines, which are associated with mitochondrial oxidative phosphorylation, energy metabolism, and extracellular matrix structure [14]. The data strongly suggest that Nrf2 plays a critical role in SkM function by regulating gene expression and inducing protein post-translational modification. In the present study, we hypothesized that ExT enhances SkM function via activation of Nrf2 signaling and that the beneficial adaptations of SkM to ExT are attenuated after Nrf2 gene deletion.

## 2. Materials and Methods

All animal procedures were conducted in accordance with the guidelines of the National Institutes of Health Guide for the Care and Use of Laboratory Animals and conformed to ARRIVE Guidelines (https://www.nc3rs.org.uk/arrive-guidelines, accessed on 9 September 2021), as approved by the Animal Care and Use Committee of the University of Nebraska Medical Centre (UNMC-IACUC Protocol #18-174-02).

### 2.1. Animal Preparation

Forty-eight male iMS-*Nrf2^flox/flox^* mice between the ages of 12 and 20 weeks were used in the present study. The iMS-*Nrf2^flox/flox^* model is a Tamoxifen inducible- skeletal muscle specific- Nrf2 knockout model created in our laboratory by crossing the HSA-MCM line [15] (Jackson Laboratory Stock No. 025750) with a Nrf2*^flox/flox^* line [16] (provided by Dr. Shyam Biswal of Johns Hopkins University, Baltimore, USA). This model has been previously tested and validated by genotyping, RT-PCR, and Western blotting and characterized by proteomic and bioinformatic analysis [13]. Mice were assigned to four groups (12/group): wildtype sedentary (WT-Sed), wildtype exercise training (WT-ExT), Nrf2 knockout sedentary (KO-Sed), and Nrf2 knockout exercise training (KO-ExT). In the KO groups, the skeletal muscle Nrf2 gene was inactivated by intraperitoneal injections of Tamoxifen at the age of 12 weeks for five consecutive days (2 mg/0.2 mL day^−1^, Sigma-Aldrich, St. Louis, MO, USA; Cat. No. T5648). Control mice (Nrf2-intact control) received vehicle administration (15% ethanol in sunflower seed oil, 0.2 mL day^−1^ for 5 days). Four weeks post Tamoxifen, muscle Nrf2 gene KO mice and controls underwent the following experiments.

### 2.2. In Vivo Mouse Experiments

*Exercise training*—Mice in ExT groups received three consecutive weeks of training on a treadmill without incline as previously described [17], with a slight modification. The training protocol consisted of one warm-up period at the beginning and one cool-down period at the end of the training session at a speed of 6 m/min for 5 min, between which was a 50 min training period at 60% maximal workload. The maximal workload was calculated using the protocol described in the following section. Due to significant differences in initial exercise capacity among groups, the training intensity was set at 14 m/min for the WT-ExT group and 9 m/min for the KO-ExT group.

*Exercise capacity evaluation*—All mice received two tests (before and after ExT/Sed interventions) for maximal running distance on a treadmill (Model no. Exer-3/6 Treadmill; Columbus Instruments, Columbus, OH, USA), as previously described from our laboratory [18]. Over the first three days, the mice were placed on the treadmill for 20 min/day without running to acclimatize them to the test environment. On day 4, the treadmill was run at an inclination of 15° with the rear shocking grid on, starting at a speed of 6 m/min for 6 min, followed by an increase of 3 m/min every 3 min until exhaustion. Exhaustion was defined when the mice remained on the shocking grid (0.2 V, 1 Hz) for 5 s without attempting to reengage the treadmill.

*In Situ Muscle Experiments*—In situ tibialis anterior (TA) contractility was examined for both muscle force generation and fatigue tolerance, according to previous methods used on soleus and extensor digitorum longus muscles in our laboratory [18]. Under 2% isoflurane anesthesia, mice were placed on a metal heating pad in the supine position. A small incision on the right anterior ankle skin was made where the TA tendon was identified, severed, and tied with a no. 6 silk suture attached to a force transducer (MLT1030/A, ADInstruments; Colorado Springs, CO, USA). A silver bipolar electrode was then inserted into the right TA belly and an intermittent tetanic stimulation with trains of square wave pulses (2.5 V, 0.3 s at 50 Hz per 3 s for a total of 20 min) was delivered by a pulse generator (A310, WPI; Sarasota, FL, USA). Force generation was determined by averaging the force of the initial three tetanic contractions. Fatigue tolerance was then calculated as the percent force generated by the last three tetanic contractions to the initial three tetanic contractions. During the experiment, the mice were kept warm using an isothermal pad and heat lamp, while the TA belly and tendon were moisturized by periodic administration of warm saline. Once the functional assessment was completed, the mice were euthanized by inhalation of CO_2_. The left TA belly was collected, snap-frozen in liquid nitrogen, and then saved in a −80 °C freezer for the following ex vivo analyses. We chose the left, rather than right, TA muscle for molecular biological assays to avoid potential contraction-induced influences on protein expression.

### 2.3. Ex Vivo Experiments

*Mass spectrometry-based proteomics*—Proteomic analysis of TA muscle was carried out as previously described [13]. Briefly, 30 mg muscle mass/sample was homogenized in Pierce RIPA buffer (Thermo Scientific, Rockford, IL 61101, USA) with 1% protease inhibitor cocktail (Abcam, ab65621) and then centrifuged at 20,000× *g* 20 min at 4 °C for protein extraction. Each sample produced 100 µg protein, which was equally distributed for proteomic analysis and Western blot assay. 50 µg protein was first reduced and alkylated with DTT and iodoacetamide respectively, following removal of detergent using chloroform/methanol extraction. The protein pellet obtained was re-suspended in 50 mM ammonium bicarbonate and digested with MS-grade trypsin to obtain peptides, which were re-suspended in 2% acetonitrile and 0.1% formic acid. 500 ng of peptide was loaded onto a trap column (Acclaim PepMap 100 75 μm × 2 cm C18 LC, Thermo Scientific, Rockford, IL 61101, USA) at a flow rate of 4 μL min^−1^ then separated with a Thermo RSLC Ultimate 3000 (Thermo Scientific) on a separate column (Thermo Easy-Spray PepMap RSLC C18 75 μm × 50 cm C-18 2 μm, Thermo Scientific, Rockford, IL 61101, USA) with a step gradient of 4–25% solvent B (0.1% FAin 80%ACN) for 10–130 min and 25–45% solvent B for 130–145 min at 300 nl min^−1^ and 50 °C. Eluted peptides were analyzed by a Thermo Orbitrap Fusion Lumos Tribrid (Thermo Scientific) mass spectrometer in a data-dependent acquisition mode. A survey full scan MS (*m*/*z* 350–1800) was acquired in the Orbitrap with a resolution of 120,000. The automatic gain control (AGC) target for precursor ion scan (MS1) was set as 4 × 10^5^ and ion filling time set at 100 ms. The most intense ions with charge state 2–6 were isolated in 3 s cycles and fragmented using higher energy collisional dissociation fragmentation with 35% normalized collision energy and detected at a mass resolution of 30,000 at 200 *m*/*z*. The AGC target for MS/MS was set at 5 × 10^4^ and ion filling time at 60 ms; dynamic exclusion was set for 30 s with a 10 ppm mass window. Label free quantitative analysis was performed using progenesis QI proteomics 4.2 (Nonlinear Dynamics, Milford, MA, USA). Individual raw files for biological replicates (n = 6) for each condition were imported and automatically aligned to a reference standard run by the software. A threshold alignment score of 75% and above was chosen as a cut off value. After peak picking, all the detected features were normalized to a reference run followed by ion abundance quantification. Quantified peaks were exported and searched for peptide identification against the Swiss-prot *Mus musculus* protein database downloaded on 13 February 2019 using the in-house mascot 2.6.2 (Matrix Science, Boston, MA, USA) search engine. The search was set up for full tryptic peptides with a maximum of two missed cleavage sites. Acetylation of protein N-terminus and oxidized methionine were included to serve as variable modifications; carbamidomethylation of cysteine was set as fixed modification. The precursor mass tolerance threshold was set to 10 ppm and the maximum fragment mass error was 0.02 Da. The significance threshold of the ion score was calculated based on a false discovery rate of 1%. Qualitative analysis was performed using protein expression fold changes between WT-ExT vs. WT-Sed and KO-ExT vs. KO-Sed were represented as Log_2_FC^WT-ExT^ and Log_2_FC^KO-ExT^, respectively.

*Western blot analysis*—Western blotting was then employed to confirm hits identified from the proteomics screen. Briefly, 50 µg protein/sample was boiled for 5 min and then loaded on a 7.5% SDS-PAGE gel (30 µg protein/10 µL per well) following electrophoresis using a Bio-Rad mini gel apparatus at 40 mA/gel for 45 min. The fractionated protein on the gel was stained by Ponceau S and then electrically transferred onto a polyvinyl difluoride membrane (Millipore). The membrane was first probed with following antibodies: NQO1 (ab80588, Abcam), SOD2 (sc-30080, Santa Cruz Biotechnology, Dallas, Texas 75220, USA), GSTM1 (12412-1-AP, Proteintech, Rosemont, IL60018, USA), and GSTM3 (15214-1-AP, Proteintech). The secondary antibodies were HRP Goat Anti-Rabbit IgG and HRP Goat anti-Mouse IgG (Thermo-Fisher Scientific, Rockford, IL 61101, USA). After three washes with TBST, the membrane was treated with an enhanced chemiluminescence substrate (Pierce; Rockford, IL, USA) for 5 min. The blots on the membrane were visualized and analyzed using a UVP BioImaging System (EpiChemi II Darkroom). The final reported data were normalized through Ponceau S staining.

*Differential proteomic and pathway enrichment analyses*—Proteins identified by mass spectrometry were quantified to identify differentially expressed proteins between each experimental and control condition. ANOVA *p*-value and absolute fold changes were used to identify differentially expressed proteins between sedentary and training mice. A protein was considered to be differentially expressed if the *p*-value was ≤0.05 and the absolute fold change ≥1.5. Gene enrichment analyses of differentially regulated proteins to identify known functions, pathways, and networks affected were performed using Ingenuity Pathway Analysis (IPA) (Ingenuity Systems; Mountain View, CA, USA).

*Dihydroethidium (DHE) Staining*—DHE staining was employed to evaluate muscle ROS. The unfixed frozen mouse TA muscle was cut into 30-μm sections using a cryostat (Leica CM 1850) and placed on glass slides, which were immersed in 2 × 10^−6^ mol/L DHE diluted with DMSO and acetone. After incubation in a light-protected, humidified chamber at 37 °C for 30 min, extra staining solution was removed, followed by three rinses with PBS. The fluorescence generated by oxidized DHE was detected using a laser confocal microscope (Leica TSC STED) using 488-nm excitation wavelength and a 585-nm filter. The relative fluorescent intensity of image was quantified using Image-J software (National Institutes of Health).

### 2.4. Statistical Analyses

*Physiological and western blot data*—Data are expressed as mean ± SD. A *t*-test was used for analyzing the differences between gene knockout mice with WT using SigmaPlot software. A *p* value of <0.05 was statistically significant.

*Proteomic and bioinformatic analyses*—For all comparisons, the ANOVA *p*-value was used. The cut-off used for the differential expression analysis summary was *p* ≤ 0.05, and for the absolute fold change, the cut off was ≥1.5. IPA pathway analysis was also performed on genes with the same cut-off. For Volcano plots, the cut-off used to add gene names to differentially expressed proteins were absolute Log2Fold change >1 and ANOVA *p*-value ≤ 0.05. For each replicate, we identified proteins that exhibit statistically significant changes in expression based on Benjamini–Hochberg (BH) method adjusted *p*-values at FDR of 0.05 to account for multiple comparisons, and volcano plots were generated using Partek Genomics Suite 7.0 (Partek Inc. Chesterfield, MO 63005, USA).

## 3. Results

### 3.1. Exercise Capacity and In Situ TA Contractility

**Figure 1:**
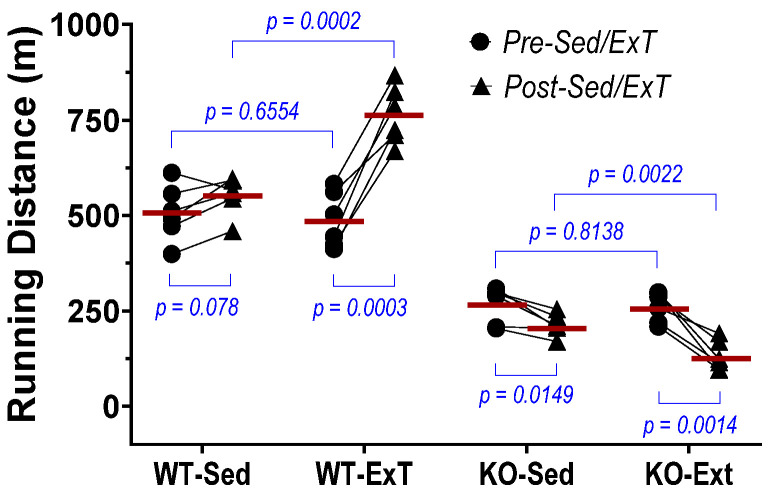
Effects of ExT on exercise performance of mice with SkM-Nrf2 intact (WT) and deleted (KO). n = 6/group.

Exercise capacity was determined by measuring maximal running distance on a treadmill at two time points, pre- and post-ExT/Sed period, in all groups (Figure 1). There was no difference at the first test (pre-ExT/Sed) within WT groups (ExT vs. Sed) or KO groups (ExT vs. Sed). In the second test, ExT-WT ran significantly further than Sed-WT (763.3 ± 74.9 vs. 552.8 ± 49.9 m, *p* < 0.001; n = 6/group), suggesting improved exercise performance following ExT when SkM-Nrf2 was intact. In contrast, when SkM-Nrf2 was deleted, there was significant impairment in exercise performance (ExT-KO 132.1 ± 39.5 vs. Sed-KO 213.7 ± 28.8 m, *p* < 0.01; n = 6/group), suggesting a critical role for Nrf2 in muscle adaptation to exercise. Surprisingly, in KO-Sed mice, we found a slightly—but significantly—reduced running distance after 3 weeks of sedentary activity. These data suggest that Nrf2 is, in fact, essential for the normal function of SkM.

**Figure 2:**
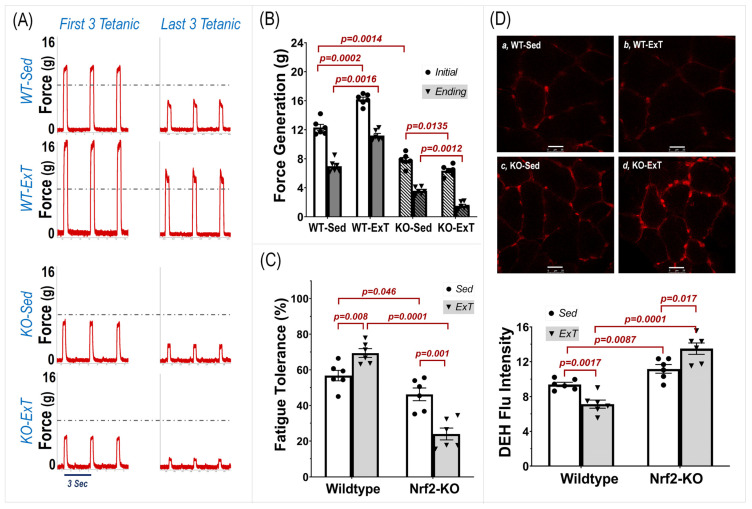
Effects of ExT on tibialis anterior contractility and ROS production in WT and SkM-Nrf2 KO mice. Contraction was induced by intermittent tetanic stimulation (2.5 V, 0.3 s at 50 Hz per 3 s for 20 min). (**A**) Original recording of the first and last three tetanic contractions. (**B**) Group data of force generation and (**C**) fatigue tolerance. (**D**) ROS level measured by DHE staining; Scale bar = 25 µm. n = 6/group.

Right TA contractility was induced by electrically stimulating muscle in situ for 20 min to determine force generation (g) and fatigue tolerance (% of ending force to initial force) (Figure 2A–C). The WT-ExT group displayed a significantly greater initial force generation (ExT-WT 16.18 ± 0.77 vs. Sed-WT 12.32 ± 1.06 g, *p* < 0.001; n = 6/group) and greater fatigue tolerance (ExT-WT 69.37 ± 5.42 vs. Sed-WT 56.78 ± 6.59 %, *p* < 0.01; n = 6/group) compared with WT-Sed mice. These data support the view of an enhancement by ExT on muscle contractility. However, these two physiological parameters were reduced in KO mice undergoing ExT (Force Generation: ExT-KO 6.37 ± 0.74 vs. Sed-KO 7.82 ± 0.92 g, *p* < 0.05; Fatigue Tolerance: ExT-KO 23.97 ± 7.48 vs. Sed-KO 46.24 ± 7.95 %, *p* < 0.001; n = 6/group), suggesting a critical role of Nrf2 in muscle adaptation to exercise. Correspondingly, ROS level was reduced in WT-ExT group (7.13 ± 0.47 vs. 9.39 ± 0.25, *p* < 0.005) but increased in KO-ExT group (13.49 ± 0.65 vs. 11.18 ± 0.49, *p* < 0.05) as compared to their Sed controls (Figure 2D).

### 3.2. Proteomics Analyses

**Figure 3:**
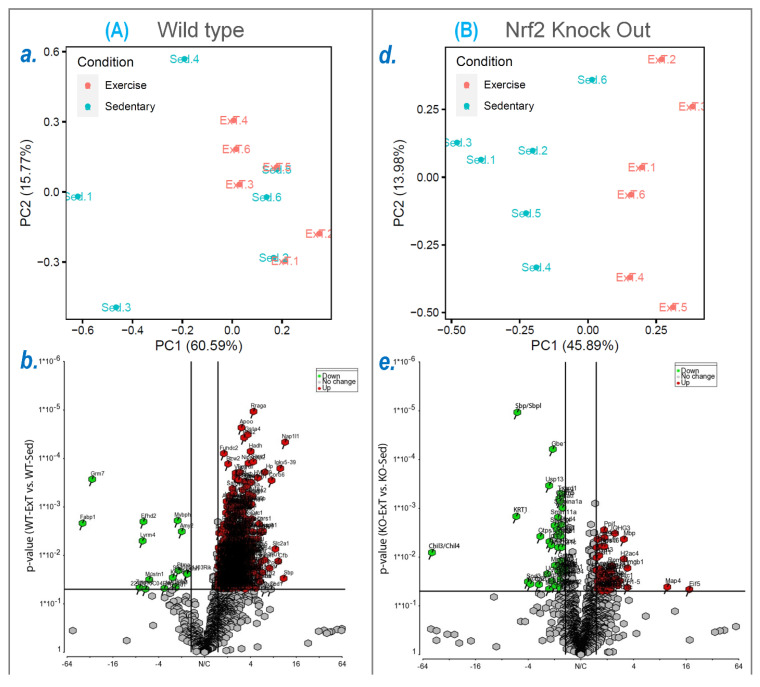

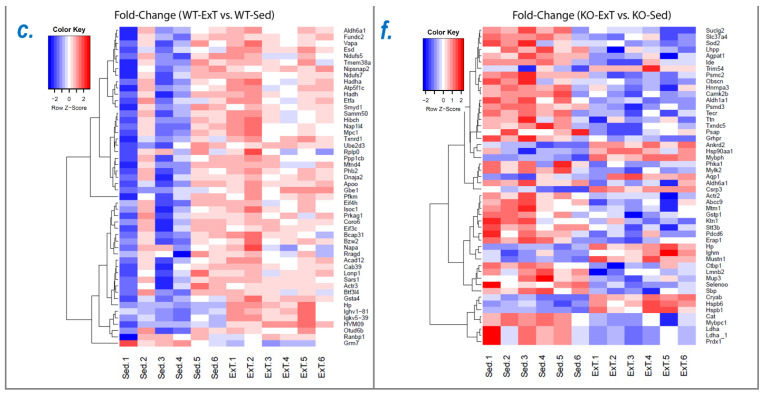
Differentially expressed proteins in TA muscles detected by mass spectrometry-based proteomics analysis with comparing WT-ExT to WT-Sed (**A**) and KO-ExT to KO-Sed (**B**) mice. (**a**,**d**) principal component analysis (PCA). The percent variance explained by each principal component is indicated on the PCA plot axes. (**b**,**e**) Volcano plots showing the log2 fold change (ExT/Sed) plotted against the –log10 *p* value, highlighting significantly changed proteins (red and green; *p* < 0.05 and an absolute fold change of 1.5, n = 6/group, moderated *t*-test). The vertical lines correspond to the absolute fold change of 1.5, and the horizontal line represents a *p* value of 0.05. Log_2_ fold changes in WT-ExT and KO-ExT are represented as Log2FC^WT-ExT^ and Log2FC^KO-ExT^, respectively. (**c**,**f**), heatmaps show the log2 of the top 50 differentially expressed proteins.

The protein profile of the TA was determined by LCMS-based label-free quantitative mass spectrometry. From the samples of WT mice, we identified and quantified 2008 proteins, of which 592 (29.48%) were significantly altered in their expression by ExT (576 upregulation and 16 downregulation; Supplementary Table S1). In the samples from KO mice, 2055 proteins were identified and quantified with 279 proteins (13.58%) expression significantly changed by ExT (72 upregulation and 207 downregulation; Supplementary Table S2). This dataset demonstrates that the predominant effect of ExT on SkM at the molecular level is to upregulate protein expression, most of which was abolished when Nrf2 was deleted. This suggests that Nrf2 plays an important role in mediating the molecular adaptation of SkM to exercise. Principal component analysis (PCA) demonstrated that separation occurred between ExT and sedentary samples with 60.59% in WT and 45.89% in Nrf2-KO of variation explained by PC1 (Figure 3a,d). Volcano plots (Figure 3b,e) indicate significantly upregulated (red dots) or downregulated (green dots) proteins following ExT with fold changes and the expression profiles of unique and overlapping proteins between ExT and Sed groups (WT-ExT vs. WT-Sed and KO-ExT vs. KO-Sed). Heatmap analysis of the top 50 protein profiles revealed a relatively good consistency of protein profiles between Sed and ExT in WT and Nrf2-KO mice (Figure 3c,f).

### 3.3. Canonical Pathway Analyses

**Figure 4:**
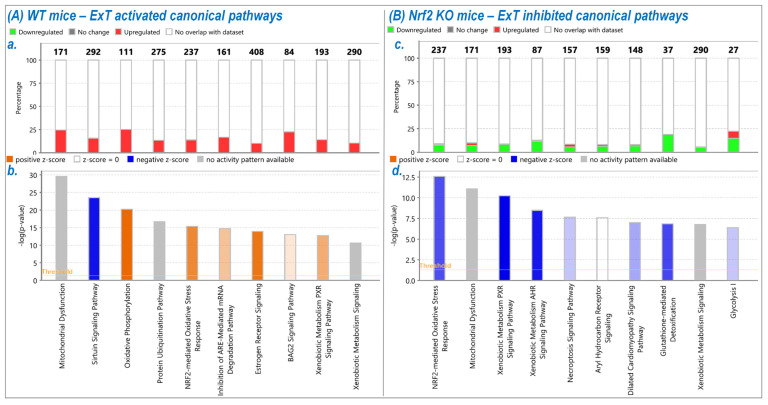
Stacked bar charts displaying the top 10 canonical pathways found to be differentially represented in comparing protein expression between ExT and Sed groups of WT mice (**A**) and Nrf2-KO mice (**B**). The bars are sorted so that the most significant pathways are at the left of the chart. (**a**,**c**) The total number of genes in each pathway is displayed above the bar. Percentage was calculated by dividing the number of identified genes by the total number of genes that map to the same pathway. (**b**,**d**) The pathway name is indicated under bar. The significance *p*-values are calculated by the right-tailed Fisher’s Exact Test, such that taller bars equate to increased significance.

**Figure 5:**
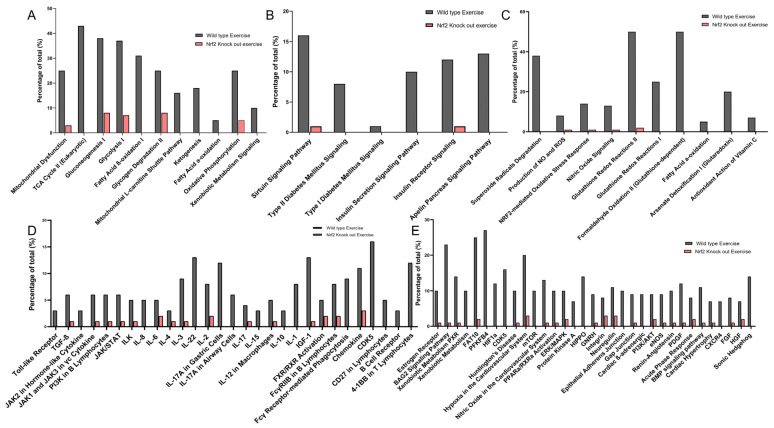
Percentage protein expression in pathways related to (**A**) energy production, (**B**) insulin signaling, (**C**) redox status, (**D**) immune signaling, and (**E**) other physiological pathways in WT-ExT mice in comparison to Nrf2-KO mice after ExT. Gray bars indicate proteins upregulated in WT mice and red bars indicate their downregulated expression levels in Nrf2-KO mice.

By performing Ingenuity Pathway Analysis (IPA) of the above proteomic profiles, we found that ExT changed 56 intracellular pathway statuses (54 activations and 2 inhibitions) in WT mice, such as the sirtuin signaling pathway, oxidative phosphorylation, Nrf2-mediated oxidative stress responses, NRE-mediated mRNA degradation pathway, HIF1a signaling, and others (Figure 4A). On the contrary, in Nrf2-KO mice, we found that ExT altered the status of 34 pathways (30 inhibitions and 4 activations), such as Nrf2-mediated oxidative stress responses, xenobiotic metabolism PXR signaling pathway, xenobiotic metabolism AHR signaling pathway, necroptosis signaling pathway, dilated cardiomyopathy signaling pathway, glutathione-mediated detoxification, and others. Figure 4 shows the top 20 signaling pathways modified by ExT in WT mice (Panel A) and Nrf2-KO mice (Panel B). This shows the total gene number/identified gene percentage for each pathway indicated in Figure 4A(a) and the full name/significance *p*-value for each pathway indicated in Figure 4A(b). The entire canonical pathway analysis data are shown in Supplementary Tables S3–S6. A comparative account of different signaling pathways involved in energy production, regulation of redox status, immune signaling, and pathways involved in general physiology in WT-ExT and in the KO-ExT mice are shown in Figure 5. Expression of proteins involved in important signaling mechanisms showed a significant reduction in Nrf2 KO mice after ExT suggesting the importance of an intact Nrf2 system in exercise mediated benefits.

### 3.4. Nrf2 Specific Pathway Analyses

**Figure 6:**
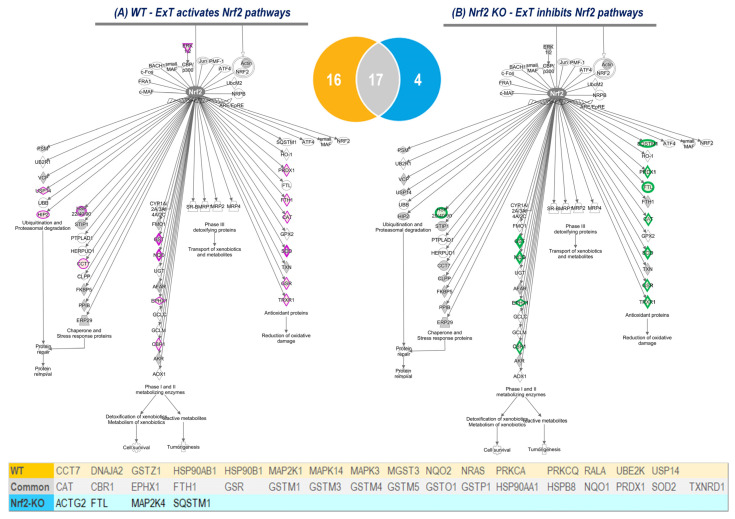
Nrf2 downstream proteins upregulated in WT mice (Purple symbols in (**A**)) and downregulated in Nrf2-KO mice (Green symbols in (**B**)) after ExT. The Venn diagram shows overlap of Nrf2-associated proteins identified in WT and Nrf2-KO mice. Their gene IDs are listed in the inserted table with yellow as WT unique, grey as the common, and blue as Nrf2-KO unique.

While IPA revealed approximately 90 intracellular pathways altered by ExT, our interest was focused primarily on Nrf2-mediated signaling. In WT mice, ExT upregulated 33 of 237 known Nrf2-associated proteins (14%; Figure 4A(a); Supplementary Table S3), whereas in Nrf2-KO mice, ExT downregulated 18 (8%) and upregulated only 3 (1%) of 237 Nrf2-proteins (Figure 4B(c); Supplementary Table S5). These results are shown in Figure 6 where it can be seen which Nrf2 target proteins were upregulated by ExT in WT mice (Purple symbols in Figure 4A) and downregulated by ExT in Nrf2-KO mice (Green symbols in Figure 4B). While the majority of these Nrf2-target proteins overlap between the two groups (see Venn Diagram), several proteins were identified only in WT or Nrf2-KO mice. These include USP14, HIP2, CCT7, FTL, FTH1, and SQSTM1, most of which are involved in ubiquitination, proteasomal degradation, protein repair, and protein removal (inserted Table in Figure 6).

### 3.5. Mitochondrial Function and Oxidative Phosphorylation Analyses

**Figure 7:**
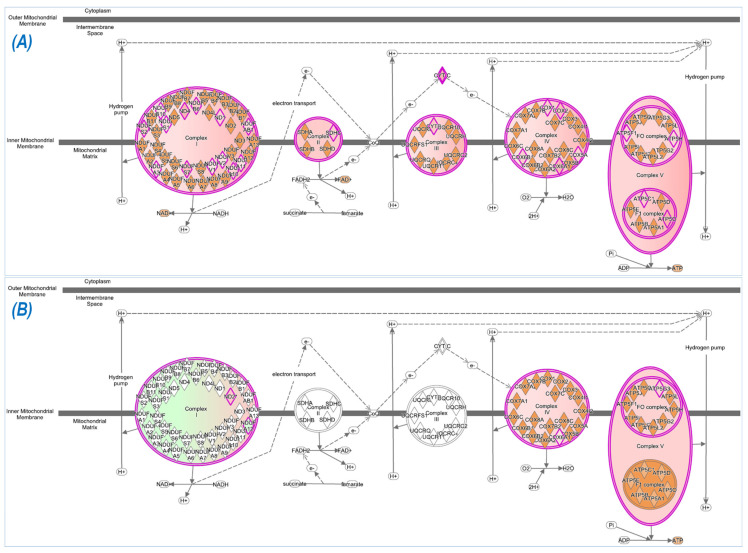
Mitochondrial function and oxidative phosphorylation in WT (**A**) and Nrf2 KO (**B**) mice following ExT. The purple outlined mitochondrial complexes with orange or green tinting are predicated to be functionally enhanced or inhibited, respectively.

One of the most important adaptations to endurance training is an increase in process of ATP production in skeletal muscle. We therefore performed the IPA analysis of proteomics data to determine ExT-induced mitochondrial function and oxidative phosphorylation. As can be seen in Figure 7, all of the five complex functions were enhanced in WT mice after ExT (Figure 7A). However, in Nrf2-KO mice, ExT reduced complex I function, enhanced complex IV and V function, but did not change complex II and III function (Figure 7B).

### 3.6. Western Blotting Analysis

**Figure 8:**
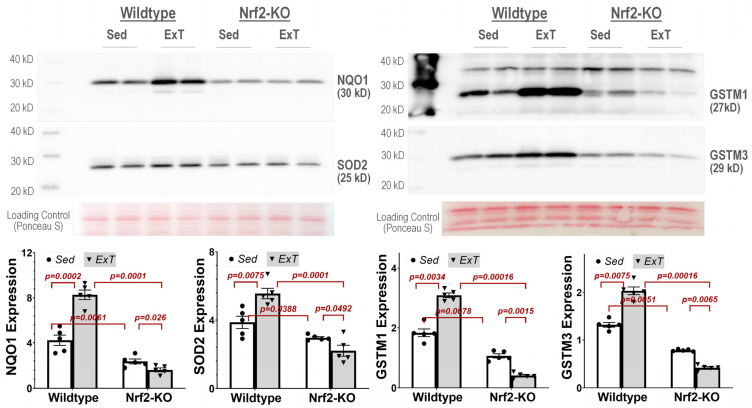
Western blotting shows that NQO1, SOD2, GSTM1, and GSTM3 are upregulated in WT-ExT and downregulated in KO-ExT as compared to Sed controls. n = 5/group.

Western blotting was utilized to validate the sensitivity and accuracy of mass spectrometry by evaluating the expression levels of NQO1, SOD2, GSTM1, and GSTM3—four principal antioxidant enzymes selected from Figure 6 which were upregulated in WT mice and downregulated in Nrf2-KO mice following ExT. As can be seen from Figure 8, in WT mice all proteins were dramatically upregulated following ExT (NQO1: 8.28 ± 0.43 vs. 4.24 ± 0.46, *p* = 0.0002; SOD2: 5.55 ± 0.31 vs. 3.86 ± 0.36, *p* = 0.0075; GSTM1: 3.09 ± 0.08 vs. 1.83 ± 0.13, *p* = 0.0034; GSTM3: 2.03 ± 0.08 vs. 1.32 ± 0.05, *p* = 0.0075. n = 5/group). In KO-Sed mice however, expression of these proteins was lower as compared with WT-Sed mice, which was further downregulated following ExT (NQO1: 1.62 ± 0.16 vs. 2.36 ± 0.22, *p* = 0.026; SOD2: 2.19 ± 0.32 vs. 2.96 ± 0.07, *p* = 0.049; GSTM1: 0.41 ± 0.04 vs. 1.06 ± 0.07, *p* = 0.0015; GSTM3: 0.42 ± 0.02 vs. 0.78 ± 0.01, *p* = 0.0065. n = 5/group). These changes were consistent with the mass spectrometry data shown in Figure 6.

## 4. Discussion

As a master antioxidant transcription factor, Nrf2 is known to regulate muscle metabolism, function, and structure under both physiological and pathophysiological conditions [19,20]. Using proteomic and bioinformatic analyses in muscle specific Nrf2 knockout and overexpression mouse lines, iMS-*Nrf2^flox/flox^* and iMS-*Keap1^flox/flox^*, we previously identified over 200 Nrf2-targeted proteins in muscle, which were actively involved in oxido-reduction coenzyme metabolism, purine ribonucleoside triphosphate metabolism, ATP metabolism, propanoate metabolism, cellular detoxification, NADP metabolism glutathione metabolism, electron transport chain, and other functions [13]. Using a redox proteomics technique, we further quantified 34 cysteine redox peptide-bearing proteins, which were associated with mitochondrial oxidative phosphorylation, energy metabolism, and extracellular matrix [14]. On the other hand, in a permanent coronary artery ligation-induced heart failure mouse model with reduced ejection fraction (HFrEF), we showed a markedly weakened Nrf2/antioxidant defense, impaired muscle function/structure, and reduced exercise capacity, which were ameliorated by the Nrf2 activator curcumin [18]. Because of these crucial roles of Nrf2 in SkM, we hypothesized that during exercise, the internal environment of myocytes is disturbed by contraction-induced multiple stressors including accumulated ROS, in which Nrf2 is activated and functions as a hub to maintain cellular homeostasis by upregulating antioxidant enzymes and other cytoprotective proteins. This Nrf2-mediated response underpins SkM adaptation to exercise and exercise-associated benefits.

We found a significant increase in maximal running distance in WT mice after 3-weeks of training, while this exercise capacity did not change in 3-week sedentary mice as a time course control. On the other hand, evaluation of TA contraction further indicated that both force generation and fatigue tolerance were significantly increased in ExT mice compared with non-ExT controls. The data from the in vivo and in situ experiments suggest that ExT markedly promotes treadmill running performance that is, at least in part, due to enhanced muscle contractility and other functions. In contrast with WT mice, SkM-Nrf2 deficient mice showed a significantly shorter running distance after ExT, which accompanied a markedly reduced muscle force generation and impaired fatigue tolerance. This evidence strongly suggests that Nrf2 plays a crucial role in exercise-induced beneficial adaptation in SkM. Without Nrf2-supported antioxidant defense and cytoprotective mechanisms in response to increased oxidative stress during exercise, the ROS surge that accompanies muscle contraction tends to damage skeletal myocytes. Furthermore, we found that exercise performance of mice with Nrf2 deficiency was reduced, even in the 3-week sedentary period (Post-Sed vs. Pre-Sed in KO-Sed group, Figure 1), suggesting that a compensatory mechanism to Nrf2-deletion was not apparent at week 3 after Nrf2 was deleted by Tamoxifen administration. However, due to technical limitations, we were not able to determine if the in-situ TA contractility in the same Nrf2-KO mouse was further impaired after 3-weeks in sedentary conditions.

To explore the molecular mechanisms underlying functional alterations after ExT and the role of Nrf2 deletion, we utilized mass spectrometry to determine the protein profile of the TA, followed by downstream bioinformatic analysis to reveal signaling pathways and integrative networks involved under these conditions. In WT mice, we identified 576 proteins in TA that were upregulated by ExT, most of which are newly reported to be associated with exercise (Supplementary Table S1). The protein with the greatest increase was adenylyltransferase (gene name: SELENOO), which was expressed at a very low level in Sed muscle, but was 8211-fold upregulated after exercise (3651.64 ± 760.45 vs. 231.98 ± 87.77, *p* < 0.0062; n = 6/group). This enzyme catalyzes transfer of adenosine 5′-monophosphate (AMP) to Ser, Thr, and Tyr residues of target proteins and results in protein adenylylation (AMPylation), playing a role in protein post-translational modification [21]. There are two protein AMP transferases. We identified the highly conserved pseudokinase selenoprotein-O (SELO) located inside mitochondria [22]. SELO plays a critical role in the response to oxidative stress and regulates global S-glutathionylation levels via AMPylation in conjunction with glutaredoxin [23]. However, its functional significance in SkM and exercise remains to be determined. Another interesting protein we identified was haptoglobin, whose expression was high in muscle tissues even under basal conditions and was subsequently upregulated 6-fold after ExT (1,014,955.2 ± 200,279.7 vs. 158,233.3 ± 9191.8, *p* < 0.0062; n = 6/group). This protein has been demonstrated to play a key function in SkM to prevent oxidative stress/inflammation and maintain muscle mass/function [24]. Absence of haptoglobin causes muscle atrophy and weakness due to the activation of an atrophic program, which could be further exacerbated by acute exercise or high fat diet [24]. A recent study showed that intravenous infusion of exogenous haptoglobin in rats attenuates hemoglobin-impaired muscle contraction, and it is therefore recommended for improved muscle metabolic control and exercise tolerance in patients suffering from acute or chronic hemolysis [25]. Other studies indicate that exercise induces global histone modifications which mediate chromatin remodeling and transcriptional activation in SkM [26,27,28].

Exercise trained wild type mice also showed upregulated levels of SLC2A1, (also referred as Solute Carrier Family 2 Member 1). Abnormal expression of this protein demonstrates various neurological dysfunctions such as Dystonia 9, Stomatin-Deficient Cryohydrocytosis, and Glucose transporter type 1 deficiency syndrome (patients suffer from severe body movement disorders and developmental and varying degrees of cognitive impairments [29,30,31]).

In SkM-Nrf2 KO mice, we found that 207 proteins were downregulated following ExT (Supplementary Table S2). The highest downregulated protein was chitinase-3-like protein 1, a glycoprotein consisting of 383 amino acids with a molecular mass of 40 kDa, which counteracts TNFα-mediated inflammation and insulin resistance in SkM cells [25]. Additionally, one of the Nrf2 downstream target proteins, glutathione S-transferase Mu 5 (Gstm5) was listed as the one of the top 6 downregulated proteins in SkM with Nrf2 deletion after exercise. This protein belongs to one of eight GST supergene families: the GSTAs, GSTMs, GSTTs, GSTP, GSTZ, GSTS, GSTK, and GSTO, located on seven chromosomes [32]. It has been reported that Gstm5 expression was significantly downregulated in ovarian cancer [33] and lung adenocarcinoma [34]. In colorectal cancer, the expression of Gstm5 was reinforced by miR-20b-3p and actively regulates miR-20b-3p/GSTM5/AKT-mTOR signaling axis [35].

Using IPA analysis of the protein profiles, we found that the functional status of 56 intracellular signaling pathways in WT and 34 pathways in KO mice were significantly modified by ExT. The most noticeable of these was the Nrf2-mediated oxidative stress response, which was accentuated in WT but suppressed in KO mice following exercise, clearly suggesting a critical role of Nrf2 in ExT-evoked muscle adaptation. We have previously reported a reduction in Nrf2 signaling in SkM when Nrf2 was knocked out [13,14]. In the current study, we demonstrated that this impaired pathway was further suppressed after exercise. This negative response of Nrf2-associated signaling to exercise, is believed to be partly responsible for the ExT-induced impairment of exercise capacity and muscle contractility in mice with SkM-Nrf2 deficiency. Among the 237 proteins associated with Nrf2-mediated signaling pathways, ExT upregulated 33 proteins in WT mice and changed 21 proteins (18 downregulated and 3 upregulated) in KO mice. A total of 17 proteins were found overlapping between groups. These proteins, we propose, represent the loyal targets of Nrf2 in muscle, which are exclusively regulated by this master antioxidant transcription factor. Without the positive regulation from Nrf2, these proteins dramatically decline in SkM following ExT. In Nrf2 KO mice, ExT-induced elevation in fatty acid binding protein (FABP) expression may also indicate adrenergic overdrive, acute myocardial infarction, and ventricular tachyarrhythmia [36]. Nrf2 KO mice also showed elevated levels of proliferation and apoptosis adaptor protein 15 (PEA-15), a ubiquitously expressed small protein in different types of cells such as fibroblasts, skeletal muscle, and adipose tissues. Its overexpression has been associated with reduced insulin sensitivity, often leading to type 2 diabetes [37,38]. The signaling of PEA-15 is also actively involved in cancer development and progression [39]. Nrf2 deletion also upregulated levels of CHI3L1, which is a chitinase 3-like protein belonging to the glycosyl hydrolase family. Nrf2 has been shown to negatively regulate CHI3L1, in a post-traumatic osteoarthritis disease model [40].

In addition to Nrf2-signaling, several other antioxidant mechanisms were also activated by exercise, such as HIF1α signaling, nitric oxide signaling, glutathione-mediated detoxification, production of nitric oxide and reactive oxygen species, and glutathione redox reactions. IPA analysis also identified intracellular pathways other than those associated with antioxidant defense. These pathways were, in general, activated in WT, but inactivated in Nrf2-KO. Following ExT, these are predominantly associated with the regulation of overall physiological response—such as insulin production, immune activation, and redox status. As expected, our data demonstrated that many metabolic pathways associated with ATP generation were activated in WT mice following ExT, including oxidative phosphorylation, tricarboxylic cycle, gluconeogenesis, glycolysis, and fatty acid β-oxidation. These upregulated metabolic pathways are essential for establishing and maintaining muscle contraction during exercise, due in part to the relatively small intramuscular stores of ATP [41].

In summary, the present study demonstrated that ExT enhanced exercise performance and muscle contractility in mice. These effects were markedly abrogated when SkM-Nrf2 was deleted. We further observed that the majority of the proteins we identified and the intracellular signaling pathways were upregulated and activated in WT mice but downregulated and suppressed in KO mice following exercise. These data suggest a critical role of Nrf2 in the exercise-induced beneficial effects on SkM. In addition, these identified proteins and signaling pathways provide insight into novel mechanisms underlying SkM adaptation to exercise and a potential therapeutic strategy to treat diseases associated with myopathy.

## Data Availability

The data are contained within the article and supplementary materials.

## Supplementary Materials

The supporting information can be downloaded at: https://www.mdpi.com/article/10.3390/antiox12010151/s1.
